# Resting-State Coupling between Core Regions within the Central-Executive and Salience Networks Contributes to Working Memory Performance

**DOI:** 10.3389/fnbeh.2016.00027

**Published:** 2016-02-25

**Authors:** Xiaojing Fang, Yuanchao Zhang, Yuan Zhou, Luqi Cheng, Jin Li, Yulin Wang, Karl J. Friston, Tianzi Jiang

**Affiliations:** ^1^Key Laboratory for NeuroInformation of the Ministry of Education, School of Life Science and Technology, University of Electronic Science and Technology of ChinaChengdu, China; ^2^Key Laboratory of Behavioral Science and Magnetic Resonance Imaging Research Center, Institute of Psychology, Chinese Academy of SciencesBeijing, China; ^3^National Laboratory of Pattern Recognition, Institute of Automation, Chinese Academy of SciencesBeijing, China; ^4^Key Laboratory of Cognition and Personality (Ministry of Education), School of Psychology, Southwest UniversityChongqing, China; ^5^Wellcome Trust Centre for Neuroimaging, Institute of Neurology, University College LondonLondon, UK; ^6^Brainnetome Center, Institute of Automation, Chinese Academy of SciencesBeijing, China; ^7^CAS Center for Excellence in Brain Science, Institute of Automation, Chinese Academy of SciencesBeijing, China; ^8^Queensland Brain Institute, University of QueenslandBrisbane, QLD, Australia

**Keywords:** working memory, dorsolateral prefrontal cortex, resting state fMRI, functional connectivity, effective connectivity, spectral dynamic causal modeling

## Abstract

Previous studies investigated the distinct roles played by different cognitive regions and suggested that the patterns of connectivity of these regions are associated with working memory (WM). However, the specific causal mechanism through which the neuronal circuits that involve these brain regions contribute to WM is still unclear. Here, in a large sample of healthy young adults, we first identified the core WM regions by linking WM accuracy to resting-state functional connectivity with the bilateral dorsolateral prefrontal cortex (dLPFC; a principal region in the central-executive network, CEN). Then a spectral dynamic causal modeling (spDCM) analysis was performed to quantify the effective connectivity between these regions. Finally, the effective connectivity was correlated with WM accuracy to characterize the relationship between these connections and WM performance. We found that the functional connections between the bilateral dLPFC and the dorsal anterior cingulate cortex (dACC) and between the right dLPFC and the left orbital fronto-insular cortex (FIC) were correlated with WM accuracy. Furthermore, the effective connectivity from the dACC to the bilateral dLPFC and from the right dLPFC to the left FIC could predict individual differences in WM. Because the dACC and FIC are core regions of the salience network (SN), we inferred that the inter- and causal-connectivity between core regions within the CEN and SN is functionally relevant for WM performance. In summary, the current study identified the dLPFC-related resting-state effective connectivity underlying WM and suggests that individual differences in cognitive ability could be characterized by resting-state effective connectivity.

## Introduction

Working memory (WM) refers to the temporary maintenance and manipulation of information that is essential for higher-order cognitive processing, including comprehension, learning, and reasoning (Baddeley, 1992). Using functional imaging, researchers found that WM is associated with the prefrontal cortex, medial and inferior temporal lobes, and areas near the intraparietal sulcus (Baddeley, 2003; Owen et al., 2005; Rottschy et al., 2012), which respectively are implicated in executive functioning, episodic processing and declarative memory, and the phonological store (Baddeley, 2000, 2003; Petersson et al., 2006). Furthermore, regional activation studies reported that WM tasks consistently recruit the dorsolateral prefrontal cortex (dLPFC; linked to encoding and manipulating information), the dorsal anterior cingulate cortex (dACC; implicated in error detection and performance adjustment), and the ventrolateral prefrontal cortex (vLPFC) extending to the anterior insula (involved in retrieving, selecting information, and inhibitory control; Owen, 1997, 2000; Carter et al., 1999; D’Esposito et al., 2000; Ramautar et al., 2006; Aron et al., 2014). These task-based findings have elucidated some aspects of the functional anatomy of the WM.

Since a study revealed that resting-state activity is correlated with subsequent WM performance (Hampson et al., 2006), investigators have realized that resting-state functional magnetic resonance imaging (rs-fMRI) is a useful technique for understanding the neural basis of the WM. Subsequent studies confirmed this viewpoint by delineating the correlations between WM performance and intrinsic resting-state activity in brain regions. For example, coherent neuronal activity between the dLPFC and medial prefrontal cortex during rest was revealed to be related to WM accuracy (Hampson et al., 2010). However, complex cognitive functions are not reflected by one or two brain regions but rely on many brain regions. Specifically, the WM network comprises multiple intrinsic connectivity networks. Each of them represents a fundamental aspect of the functional brain organization, which consists of some regions that have similar functions. Therefore, increasing attention has been paid to uncovering the correspondence between the spatial composition of these core regions in the intrinsic organizations and the regions engaged by specific cognitive processes (Smith et al., 2009; Di et al., 2014b). For example, some core regions (e.g., the dLPFC, insular areas, and anterior cingulate cortex) in the intrinsic organizations (e.g., the executive-control and frontoparietal networks) identified using resting-state data have been revealed to be involved in WM (Smith et al., 2009). Another study indicated that the regional amplitudes of the resting-state activity of WM regions could predict the activity of these regions during a WM task (Zou et al., 2013).

Although these studies indicated that some brain regions captured at rest are engaged by WM ability, WM is actually achieved by cooperation between distinct regions (Baddeley, 2003). Hence, investigating the independent functions of core WM-related regions may be inappropriate for delineating the ways these regions are involved in WM. Gordon et al. (2012) attempted to address this issue by studying the spatial similarity between the WM network detected at rest and the regions activated during cognitive tasks and suggested that the patterns of activation during a WM task may result from integrating distinct WM-related regions obtained from data collected during rest. However, neither that study nor subsequent ones (Tu et al., 2012; Gordon et al., 2015) revealed the specific mechanism (such as a causal relationship) for this integration. Therefore, dynamic causal modeling (DCM; Friston et al., 2003), which is able to deduce this causal relationship at rest (Razi et al., 2015), may help to clarify this issue.

In the present study, using a large sample of healthy young adults, we investigated the functional relationship between WM ability and the interactions between core WM regions during rest. We first identified the WM-related regions by linking WM accuracy to resting-state functional connectivity (rsFC) with the bilateral dLPFC, the core region in the central-executive network (CEN) that is most frequently involved in WM (Rottschy et al., 2012). Then spectral dynamic causal modeling (spDCM) was used to quantify the effective (corrected) connections between these regions. Finally, a correlation analysis of the effective connectivity and WM accuracy was conducted to investigate the relationship between WM performance and coupling between WM-related regions.

## Materials and Methods

### Participants

Two hundred and sixty-four right-handed, healthy young adults (141 females, age: 22.7 ± 2.4 years, education in years: 15.5 ± 2.6) with no history of neurological or psychiatric disease were recruited. Nine participants who did not take the behavioral test were excluded. All participants signed a written, informed consent form that was approved by the Medical Research Ethics Committee of Tianjin Medical University.

### Data Acquisition

Rs-fMRI scanning was performed on a Signa HDx 3.0 Tesla MR scanner (General Electric, Milwaukee, WI, USA). Foam padding was used during the scanning to reduce head motion and earplugs to reduce scanning noise. Rs-fMRI data were obtained using a Single-Shot Echo-Planar Imaging sequence (SS-EPI) with the acquisition parameters as follows: no gap, 3.75 mm × 3.75 mm × 4.0 mm (voxel size), 2000/30 ms (TR/TE), 240 mm × 240 mm (FOV), 64 × 64 (resolution within slice), 90° (flip angle), 40 transverse slices, and 180 volumes. During the functional magnetic resonance imaging (fMRI) scans, individuals were instructed to keep their eyes closed and relax, move as little as possible, think of nothing in particular, and not fall asleep.

### Experimental Paradigm

The WM performance of each participant was evaluated by the 2-back task. This task has been widely used, especially in studying the neural basis of WM at rest (Kane and Engle, 2002; Tu et al., 2012; Gordon et al., 2015), since it is moderately difficult (Schmidt et al., 2009). The participants were required to press a button when a letter was the same as the letter they saw two letters before. The letter stimuli were case-sensitive and chosen from a set of 18 uppercase letters and 18 lowercase letters (all consonants except L, l, W, w, Y, and y). Each letter stimulus appeared for 200 ms, and the inter-stimulus interval was 1800 ms. There were three 2-back WM blocks. Each stimulus block consisted of 30 stimuli containing 10 targets, and was indicated by an instruction cue before each block. The number of correctly responding target letters was used as the WM accuracy. The results of a covariance analysis (*p* = 0.27) revealed that our study did not support a speed-accuracy trade-off. The WM task was performed outside the scanner, and the behavioral assessments were completed within 8 weeks of the fMRI study.

### Preprocessing

Resting-state data preprocessing was performed using Statistical Parametric Mapping (SPM12^1^). After discarding the first 10 time points to allow for magnetization equilibrium, the preprocessing steps for the remaining 170 functional scans included: (1) slice timing correction and realignment to the first volume to provide for head-motion correction; (2) normalization to Montreal Neurological Institute (MNI) space with resampling to 3 × 3 × 3 mm^3^; (3) spatial smoothing with a Gaussian kernel of 6 mm full-width at half maximum; (4) linear detrending; and (5) regressing out nuisance signals (six head motion parameters and global, cerebrospinal fluid, and white matter signals) and temporal band-pass filtering (0.01–0.08 Hz) for the rsFC analysis. Based on the estimated motion correction, 12 participants with more than 2 mm maximum displacement in any of the *x*, *y*, or *z* directions or more than 2° of angular rotation about any axis for any of the 170 volumes were excluded from further analysis. Although whether or not to remove the global signal is debated (Macey et al., 2004; Fox et al., 2009; Weissenbacher et al., 2009; Van Dijk et al., 2010; Saad et al., 2012), for our data, global scaling appeared to improve the specificity of the rsFC analysis.

### Seed-Based rsFC Analysis

The dLPFC is a key region in the CEN, which is most frequently involved in the WM (Hampson et al., 2010; Rottschy et al., 2012). Consistent with previous studies (based on rs-fMRI data) that viewed the dLPFC as representative of the CEN (Song et al., 2008; Hampson et al., 2010), the bilateral dLPFC were chosen as seed regions in this study. We followed the conventional rsFC analysis procedure. Specifically, the rsFC was analyzed using the Pearson’s correlation coefficient between the time series for each voxel of the whole brain and the average blood oxygen level dependent time series in the left or right dLPFC, which was defined as the left or right Brodmann area (BA) 46 (Zhou et al., 2007; Song et al., 2008). Fisher’s transformation was applied to the rsFC to transform the *r* values to *z* values. Thus, a whole-brain rsFC map of the bilateral dLPFC was created for each subject. A voxel-wise one-sample *t*-test for the rsFC map was performed in a group-level analysis to identify significant functional connectivity with the bilateral dLPFC (corrected to *p* < 0.05 using a cluster-level false discovery rate).

### Correlation Analysis of the rsFC Data and WM Performance

To investigate the association between the rsFC and WM performance, a correlation analysis of the rsFC and WM was performed using SPM12. Significant correlations were corrected to *p* < 0.05 using Monte Carlo simulations (Forman et al., 1995) with the parameters including: single voxel *p* < 0.01, 1000 simulations, full width at half maximum = 6 mm, cluster connection radius = 5 mm; with a mask and a resolution of 3 × 3 × 3 mm^3^. The results of this step provided empirical evidence for defining the connections of the various alternative models in the DCM model space.

### Correlation Analysis of the Effective Connectivity and WM Performance

Using DCM12 in SPM12, an spDCM analysis (Friston et al., 2014), which is an extension of DCM, was used to estimate the effective connectivity (Friston et al., 2003). This method has a number of advantages compared to some other effective connectivity methods (Penny et al., 2004; Friston, 2009, 2011a). DCM provides both neuronal and hemodynamic models. The former is based on low-order approximations to otherwise complicated equations describing the evolution of neuronal states. In the hemodynamic part of the DCM, neuronal activity gives rise to hemodynamic activity by a dynamic process described by an extended balloon model, which is a biophysical model involving a set of hemodynamic state variables, state equations, and hemodynamic parameters (Penny et al., 2004). The parameters of the equations in the neuronal and hemodynamic models encode the strength of the connections and delineate how they change under different conditions. Therefore these parameters are the objects that DCM tries to estimate (Friston, 2009). In effect, spectral DCM is based upon the same sort of convolution models used in conventional (whole brain) analyses of fMRI data. The only differences are that the convolution model is equipped with interregional connections and that the model fitting proceeds in the frequency domain (Razi et al., 2015).

The volumes of interest (VOIs) for the spDCM were identified based on the results of a correlation analysis of the rsFC data and WM performance. These VOIs were specified as binary masks. We extracted subject-specific estimates of the regional time series following the steps in previous studies that used resting-state DCM (Di and Biswal, 2014a; Kahan et al., 2014; Razi et al., 2015). The connectivity models we considered are described in the “Results” Section. The most likely generative models were identified using fixed effects Bayesian Model Selection (BMS; Stephan et al., 2009). The model parameters of the best model were used as summary statistics and entered into a correlation analysis (corrected to *p* < 0.05 using a cluster-level false discovery rate). This analysis tested for correlations between the effective connectivity and WM performance.

## Results

### Correlation Analysis of the rsFC Data and WM Performance

**Figure 1:**
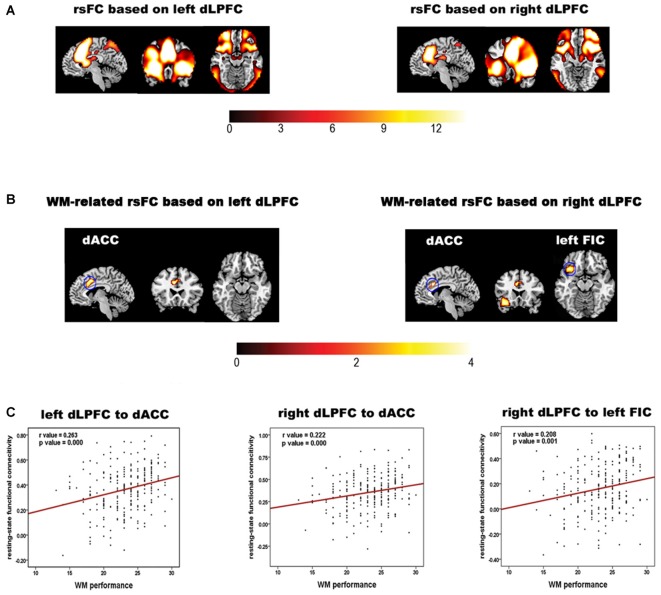
**Results of the rsFC analysis and the correlation analysis of the rsFC data and working memory (WM) performance. (A)** The bilateral dLPFCs rsFC pattern obtained by one-sample *t*-tests for the whole group. **(B)** Brain regions in which the left and right dorsolateral prefrontal cortex (dLPFC) rsFC patterns in **(A)** are correlated with WM accuracy. The color bar indicates *t* values. **(C)** Scatter plots illustrating the correlations between WM accuracy and the strength of the rsFC between the dLPFC and each brain region in **(B)**. The red line indicates that the correlation was significant (*p* < 0.05, corrected).

The rsFC maps based on the bilateral dLPFC are shown in Figure 1A. The regions showing significant correlations between the rsFC and WM performance are summarized in Table 1 and shown in Figures 1B,C. Specifically, the rsFC between the bilateral dLPFC and the dACC (Bush et al., 2000) and between the right dLPFC and the left orbital fronto-insular cortex (FIC; Seeley et al., 2007) were significantly greater than zero. Furthermore, the rsFC between the left dLPFC and the bilateral dACC was positively correlated with WM performance (*r* = 0.263, *p* = 0.000); the rsFC between the right dLPFC and the bilateral dACC was positively correlated with WM performance (*r* = 0.222, *p* = 0.000), and the rsFC between the right dLPFC and the left FIC was also positively correlated with WM performance (*r* = 0.208, *p* = 0.001). These results suggest that the stronger the rsFC between the bilateral dLPFC and the bilateral dACC and between the right dLPFC and the left FIC, the better a subject’s WM performance.

**Table 1:** **Regions with significant correlations between WM accuracy and rsFC**.

				MNI coordinate		
Seed region	Brain region	Brodmann area (BA)	Cluster (mm^3^)	*x*	*y*	*z*
Left dLPFC	Left dACC extending to right dACC	BA 32	5346	−6	15	42
Right dLPFC	Left dACC extending to right dACC	BA 24, BA 32	1215	−9	33	9
Right dLPFC	Left FIC	BA 47, BA48	3915	−36	47	13

### Spectral DCM Model Space

In a correlation analysis of the rsFC data and WM performance, the WM-related rsFC of both the left and the right dLPFC was with the same region of the dACC, although the exact range was slightly different. Therefore, the part of the dACC where the two significant correlations overlapped was selected as a VOI. In addition, we did a search based on the dACC and left FIC to explore whether there were other regions whose rsFC with the dACC or left FIC were correlated with WM performance. Specifically, we considered the dACC and the left FIC as seed regions and implemented rsFC and correlation analyses following the steps described in the “Materials and Methods” Section. However, we did not find any new VOIs. Hence, based on these results, four VOIs were used in this study—the left dLPFC, right dLPFC, dACC, and left FIC.

**Figure 2:**
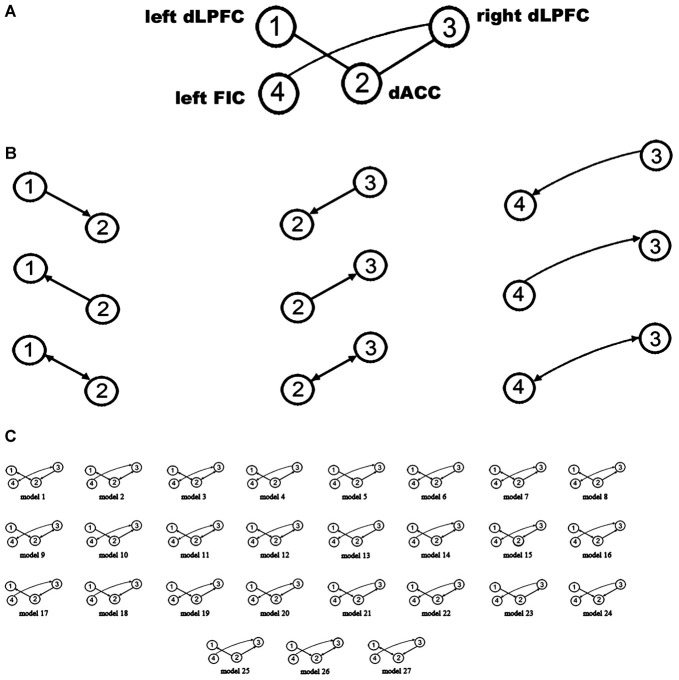
**Defining the model space. (A)** The basic architecture indicating which connections (black lines) were considered. **(B)** Possible combinations of effective connections between the bilateral dLPFC, dorsal anterior cingulate cortex (dACC), and left orbital fronto-insular cortex (FIC). **(C)** The 27 alternative models considered in the bayesian model selection (BMS). The circles indicate the VOIs used in the spDCM analysis.

A large model space containing more than 1000 models was induced by considering all combinations of the directed connections between the four VOIs. Therefore, only some (plausible) models were considered for BMS. One purpose of using DCM in our study was to attempt to explain why the rsFC between the four regions is related to WM performance. Given that a close relationship exists between effective connectivity and functional connectivity (Friston, 2011b; Friston et al., 2014; Razi et al., 2015), we constrained the plausible alternative models in DCM space based on WM-related rsFCs. Hence, after extracting the resting-state signals of the four VOIs, we employed a region-wise rsFC analysis. Subsequently, the rsFCs between these VOIs were correlated with WM performance. Only those rsFCs related to WM performance could be considered to have WM-related effective connectivity. The results of the region-wise rsFC analysis did not find any additional WM-related rsFCs other than the three connections which had been revealed in the previous steps. In other words, the WM-related effective connectivity should be considered to be between the bilateral dLPFC and the dACC and between the right dLPFC and the left FIC. Therefore, our model space focused on the connections between these three pairs of regions (Figure 2A). Furthermore, there were three possible effective connections between each pair of regions (Figure 2B). Thus, the possible combinations resulted in 3^3^ = 27 models (Figure 2C), which were examined in the spDCM analysis.

### Correlation Analysis of the Effective Connectivity and WM Performance

**Figure 3:**
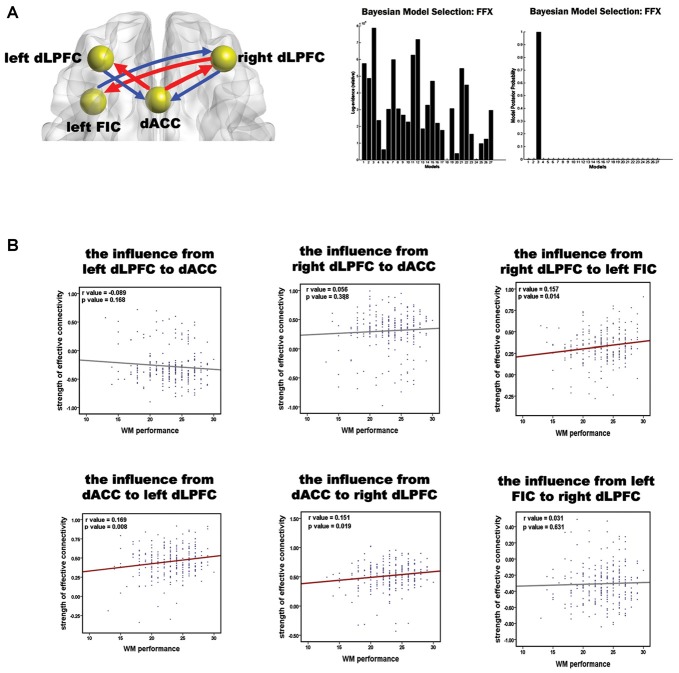
**Results of the spDCM analysis and the correlation analysis of the effective connectivity and WM performance. (A)** The structure of the optimal model and the group level BMS from 27 alternative models. In the left diagram, the spheres indicate the VOIs used in the spDCM analysis, and the red arrows represent the effective connections correlating with WM performance. The right graphs show the fixed effects BMS in terms of the log-evidence and model probability. **(B)** Results of the correlation analysis of the effective connections and WM accuracy. Red regression lines indicate that the correlation between the effective connection and WM accuracy was significant (*p* < 0.05, corrected); the gray lines indicate that the correlation was not significant.

BMS suggested that the most likely generative model was the third model (Figure 3A). This “reciprocal” model was therefore considered to be the optimal one. Table 2 shows the strength of each effective connection under this model. Supplementary *t*-tests on the effective connection strengths confirmed that the effective connectivity was extremely reliable across the subjects when tested against the null hypothesis, that is, a connection strength of zero (Table 2). The left part of Figure 3A shows the effective connectivity between the dACC and the bilateral dLPFC and between the left FIC and the right dLPFC. Although the connections were reciprocal, the effective connections that could predict WM performance were restricted to those from the dACC to the bilateral dLPFC and from the right dLPFC to the left FIC.

**Table 2:** **Statistical analysis of the resting-state effective connectivity of the winning spDCM model—the strength of the effective connections were analyzed using one-sample *t*-tests**.

Causal influence	Strength	*p* value	*t* value
Left dLPFC→dACC	−0.275 ± 0.019	0.000	−14.209
dACC→left dLPFC	0.456 ± 0.012	0.000	36.362
Right dLPFC→dACC	0.307 ± 0.021	0.000	14.327
dACC→right dLPFC	0.521 ± 0.014	0.000	36.122
Right dLPFC→left FIC	0.329 ± 0.012	0.000	27.383
Left FIC→right dLPFC	−0.307 ± 0.015	0.000	−19.921

The correlation between all the effective connections in the winning model and WM performance is shown in Figure 3B. Largely positive correlations were found between WM accuracy and the strength of the effective connections from the dACC to the left dLPFC (*r* = 0.169, *p* = 0.008), from the dACC to the right dLPFC (*r* = 0.151, *p* = 0.019), and from the right dLPFC to the left FIC (*r* = 0.157, *p* = 0.014). The three significant correlations all involved afferents to or efferents from the dLPFC.

## Discussion

The present study focused on the causal relationship between core regions of the WM network in healthy people. We found that WM performance was positively correlated with the strength of both functional and effective connectivity between core brain areas belonging to the salience network (SN; i.e., the dACC and left FIC) and the CEN (i.e., the bilateral dLPFC). Although previous WM studies frequently regarded the functional relationship between the dLPFC and dACC (Kondo et al., 2004) as well as between the dLPFC and left FIC (D’Esposito et al., 2000; Clos et al., 2014) as the neural basis of WM, they did not clarify how these two relationships interacted to function within the neural community that forms the WM system. Our work integrated the functions of the bilateral dLPFC, dACC, and left FIC and linked the dynamic causal relationship between these four regions in the resting-state to explain individual differences in the WM. Our results suggest that the interactive mechanisms of the WM can be reflected by the resting-state coupling between these WM regions.

Individual differences in WM have been suggested as relating to differences in brain connectivity, particularly in the higher order association regions (Wang and Liu, 2014). Our findings that the rsFCs of the bilateral dLPFC-dACC and of the right dLPFC-left FIC were related to WM performance support this view. In addition, our findings are highly consistent with previous studies that reported that these regions are reliably co-activated during WM tasks, especially during the 2-back WM task using letter stimuli (Cohen et al., 1994; D’Esposito et al., 2000; Kondo et al., 2004). The dACC and left FIC are core regions of the SN, which identifies the most relevant behavioral stimuli and plays an important role in cognitive control (Menon and Uddin, 2010). Existing findings (Greicius et al., 2003; Fox et al., 2006) have revealed that co-activation among these regions indicates that integration of the CEN with the SN is important for the WM. Our results extended this to the resting state and also support a recent finding suggesting that a close correspondence exists between brain activation patterns during a WM task and the engagement of core regions in multiple intrinsic organizations (Gordon et al., 2012).

BMS showed reciprocal influences between the bilateral dLPFC and the dACC and between the right dLPFC and the left FIC. Many studies, including anatomical studies and cytoarchitectural maps, have revealed a bidirectional coupling between these regions (Watson et al., 2006; Fajardo et al., 2008; Fuster, 2008; Medalla and Barbas, 2010). Most crucially, the correlation analysis in the present study revealed that, during rest, the WM performance was correlated with the dACC→dLPFC connectivity and right dLPFC→left FIC connectivity. Our findings are in accordance with a WM process that is based on a top-down mechanism (D’Esposito et al., 2000; Au Duong et al., 2005; Badre and Wagner, 2007). Specifically, the WM cognitive model (Au Duong et al., 2005) suggests that the dACC supervises the dLPFC during the WM process. This is in agreement with existing findings that revealed that the degree of activity in the dACC could predict the strength of the dLPFC activity in cognitive tasks (Kerns et al., 2004). Therefore, the dACC plays an important role in setting the activity levels of the dLPFC during the WM process (Schneider and Chein, 2003). These findings could be regarded as strong evidence for the observed dACC→dLPFC connectivity in the present study. On the other hand, another study suggested that the left FIC is recruited for preprocessing and maintaining information during the delay interval (the second stage of WM processing), whereas the dLPFC plays a crucial role in manipulating this information (D’Esposito et al., 2000). Signals from the dLPFC select the WM-relevant representation in the left FIC, thus enhancing these representations (Curtis and D’Esposito, 2003). This finding suggests the right dLPFC→left FIC connectivity identified in our study. Our findings, therefore, seem to integrate these views into one system and suggest that the directions of the information flows between these regions are responsible for the subsequent WM performance.

Our results can be further interpreted from the perspective that the DCM coupling parameters estimated from resting-state data reflect the sensitivity of a target region to its afferent signals (Kahan et al., 2014). This means that modulatory effects on coupling can be conceptualized as an afferent-specific gain modulation of the target (Kahan et al., 2014). In other words, the degree of response of the target is determined by the afferent signals. Therefore, our finding that the increased sensitivity of the bilateral dLPFC to the dACC was correlated with improved WM performance echoes a previous hypothesis (Osaka et al., 2004). In addition, our finding is in line with a recent study of rsfMRI data using a Granger causality analysis that revealed a dominant directional connection from the dACC to the dLPFC in the reciprocal connectivity between these two regions (Uddin et al., 2011). Furthermore, the correlations between better WM performance and the enhanced sensitivity of the dLPFC to the dACC is not surprising given that, as a core region in the SN, the dACC has been demonstrated to adjust temporally inappropriate responses in executive functions during WM (Schneider and Chein, 2003; Miller and D’Esposito, 2005). Specifically, many studies suggest that both the dLPFC and dACC are important regions involved with the executive system (Schneider and Chein, 2003; Barrett et al., 2004; Rueda et al., 2005). In this system, the dACC, which functions as a cognitive activity monitor, determines the level of activity that is sufficient for the executive control signals to produce a successful WM task performance (Schneider and Chein, 2003). Therefore, the interconnectivity between the dACC and the dLPFC provides a pathway through which the dLPFC can accept assistance from the dACC for mediating subsequent cognitive processes (Schneider and Chein, 2003). In summary, the enhanced sensitivity of the dLPFC to the dACC leads to a higher quality of the executive control signals. Thus, our finding suggests that a greater WM ability is related to a better self-adjustment of the executive system.

Our finding that an increased sensitivity of the left FIC to the right dLPFC subserves WM ability is interesting. This is in line with the view that the left FIC is a region that can receive causal outflow from the right dLPFC (Palaniyappan et al., 2013). It is worth mentioning that the left FIC in our finding primarily contained the vLPFC even though the FIC has usually been reported to consist of two parts: the vLPFC (BA 47/45) and anterior insula (Seeley et al., 2007). The left vLPFC has a bias towards cognitive control of memory (Badre and Wagner, 2007). For example, this region is related to the retrieval of relevant knowledge and selection of relevant representations when there is competition between active representations (D’Esposito et al., 2000; Badre and Wagner, 2007). Furthermore, the left FIC is not a strong driver of network dynamics in the CEN (Uddin et al., 2011). Hence, the influence of the left FIC on the right dLPFC should be expected to be lower than that in the reverse direction. The left FIC is a multifunctional integration region, which not only implements the integration of external and internal processes but also has a strong association with semantic and phonological processing (Clos et al., 2014). Indeed, although some findings showed that the left FIC is activated in a selection process, more studies suggested that this region may be generally involved in retrieving, encoding, and selecting abstract information from the memory (Badre and Wagner, 2007; Frings et al., 2009). The degree of this latter group of functions is determined by the demands of executive control (Engle et al., 1999; Curtis and D’Esposito, 2003). Therefore, our results suggest that a higher sensitivity of the left FIC to the dLPFC can contribute to a faster and more precise behavioral performance. In other words, the present study suggests a relationship between WM ability and the modulation of the connectivity strengths inside the executive subsystem of the WM network.

Our aforementioned result may help to clarify one issue. Specifically, in our study, the left FIC mainly involved BA 47, which is in the anterior vLPFC. Several studies suggested that the posterior vLPFC is influenced by the dLPFC and is primarily associated with the maintenance and retrieval of information in the WM (Au Duong et al., 2005). However, other studies have theoretically suggested that BA 47 appears critical in biasing these representations in the WM (Badre and Wagner, 2007). Therefore, according to our findings, it seems reasonable to suggest that BA 47 is more closely related to maintaining information. This is consistent with the hypothesis that, in a WM task, the anterior vLPFC plays a crucial role in maintaining the information manipulated by the dLPFC (D’Esposito et al., 2000).

The present work linked the dynamic interactions of the four core regions to WM ability. Furthermore, the top-down theory that best described the WM process explained why these connections reflect WM performance. Baddeley’s model suggests that WM comprises the executive system aided by two or more subsidiary slave systems (e.g., the visuospatial sketch pad, the phonological loop, and the episodic buffer; Baddeley, 2003). If we use this model, our results may elucidate the cerebral substrates of the executive control system, which is the core of the WM model. In fact, Baddeley (2003) suggested that the supervisory attentional system (Norman and Shallice, 1986) might be the basis for executive control. A subsequent study (Gazzaniga et al., 2002) attributed the attentional functions in the supervisory attentional system primarily to the anterior cingulate cortex. Therefore, consistent with our results, these findings indicated that the causal flows from the dACC to the dLPFC influence executive control. On the other hand, the left FIC has been linked to comparatively simple information processing, such as controlled access to stored conceptual representations (Badre and Wagner, 2007). Hence this region may be an important interface between the executive system and the slave systems, such as the phonological loop and the episodic buffer (Au Duong et al., 2005; Campo et al., 2012). All these findings indicated that, although the dLPFC plays a prominent role in executive control (Baddeley, 2003), executive functioning must be understood in terms of different interactions between regions belonging to different cognitive sub-networks, rather than as a specific association between one region and one higher-level cognitive process (Collette and Van der Linden, 2002). Our results are highly consistent with this view. Further, our study suggests that the degree of coupling is related to WM performance. Therefore, our findings not only demonstrated that effective connectivity during rest can predict individual differences in WM but also that it can provide novel insights into the neural substrates of WM.

There are some limitations that should be addressed for this study. First, in our work, the behavioral measure of the WM performance depended on a direct response to the 2-back WM task. Thus it is possible that other factors, for example, individual differences in responsibility, interfered with our results. To address this issue, we used the naive Bayes (reverse inference) method (Poldrack, 2006) in the BrainMap database^2^ to examine the intrinsic function of the core regions obtained in our study and found that coactivities in these regions are strongly related to executive function (Bayes factor = 22.3, meaning that the probability of the existence of this relationship is greater than 0.95). Therefore the result of reverse inference suggests that other factors may have had little influence on our observation. Second, our work paid close attention to the WM mechanism relative to the executive system, which is a central aspect of WM based on the dLPFC. Although the four regions in our work are recognized as core regions of the WM network (Owen et al., 2005; Rottschy et al., 2012), we cannot definitely conclude that the WM system can only be summarized by the interactions between these four regions. That is because, as a complex cognitive task, WM consists of many aspects or components. For instance, some studies indicated that the parietal regions are also activated in WM tasks. However, these regions were not observed in this study. This may indicate the functional segregation of the dLPFC from the parietal cortex can be captured at rest, given that the former has been intimately linked to information manipulation (D’Esposito et al., 2000; Baddeley, 2003) whereas the latter has been implicated in storing phonological short-time memory (Jonides et al., 1998; Baddeley, 2003). However further evidence is required. Therefore, studying the dissociable contributions of the dLPFC and parietal regions to WM based on rs-fMRI data should be done in future studies.

## Conclusion

Based on a large healthy Chinese sample, the present study revealed that the effective connectivity from the dACC to the dLPFC and from the right dLPFC to the left FIC was related to individual differences in WM. Our results suggest that the dLPFC is sensitive to the dACC, which can set the appropriate cognitive signal level to produce a successful WM performance. Moreover, a high sensitivity of the left FIC to signals from the dLPFC was also suggested. This increased sensitivity may help to efficiently manipulate WM-related information. These findings emphasize that this type of causal coupling between core regions in the CEN and SN is necessary for better WM performance during a WM task. Taking the abnormal co-activations of the two intrinsic connectivity networks in mental diseases (e.g., attention-deficit hyperactivity disorder and schizophrenia) into account, the present study might aid in understanding the abnormal interactions between the two networks in diseased populations with impaired WM (Silver et al., 2003; Martinussen et al., 2005).

## Author Contributions

TJ designed and supervised the research. KJF supervised DCM analysis. JL collected the data. XF, YZ, YCZ and YW analyzed the data. XF, YZ, YCZ, LC, KJF, and TJ wrote the article.

## Funding

This work was partially supported by the National Key Basic Research and Development Program (973; Grant No. 2011CB707800), the Strategic Priority Research Program of the Chinese Academy of Sciences (Grant No. XDB02030300), and the National Natural Science Foundation of China (Grant No. 91132301, 91432302, and 81101000).

## Conflict of Interest Statement

The authors declare that the research was conducted in the absence of any commercial or financial relationships that could be construed as a potential conflict of interest.

## Footnotes

^1^http://www.fil.ion.ucl.ac.uk/spm/

^2^http://www.brainmap.org

## Abbreviations

BA: Brodmann area
BMS: Bayesian Model Selection
CEN: central-executive network
DCM: dynamic causal modeling
dACC: dorsal anterior cingulate cortex
dLPFC: dorsolateral prefrontal cortex
FIC: orbital fronto-insular cortex
fMRI: functional magnetic resonance imaging
rsFC: resting-state functional connectivity
rs-fMRI: resting-state functional magnetic resonance imaging
SN: salience network
spDCM: spectral dynamic causal modeling
SPM: Statistical Parametric Mapping
vLPFC: ventrolateral prefrontal cortex
VOIs: volumes of interest
WM: Working Memory.
